# A case of traumatic thoracic aorta rupture - A life threatening emergency

**DOI:** 10.15171/jcvtr.2019.41

**Published:** 2019-05-28

**Authors:** Rupesh Kumar, Javid Raja, Ganesh Kumar Munirathinam, Anand Kumar Mishra, Rana Sandeep Singh, Shyam Kumar Singh Thingnam

**Affiliations:** ^1^Department of Cardiothoracic and Vascular Surgery, Advanced Cardiac Center, Postgraduate Institute of Medical Education and Research, Chandigarh, Pin 160012, India; ^2^Department of Cardiothoracic and Vascular Anesthesiology, Advanced Cardiac Center, Postgraduate Institute of Medical Education and Research, Chandigarh, Pin 160012, India

**Keywords:** Aorta, Aortic Transection, Aortic Rupture

## Abstract

Traumatic aortic transection is a life threatening emergency where there is a near-complete tear through all the layers of the aorta due to trauma. This condition is most often lethal and requires immediate medical attention. Symptoms of an aortic rupture may include severe chest pain, back pain, abdominal pain and signs of external chest injury. Treatment should be prompt in hemodynamically unstable patient in the form of endovascular or open surgical technique. We present a twenty nine year old male with aortic transection following motor vehicle accident where an interposition tube graft was placed after trimming the lacerated segments of the aorta under cardiopulmonary bypass. The patient is doing well with two years of follow up at our institution.

## Introduction

Traumatic aortic transection, also known as aortic rupture, is a near-complete tear through all the layers of the aorta necessitating immediate medical attention.^1^ Patients who survive to the emergency department usually have partial-thickness tears of aortic wall with pseudoaneurysm formation. When present, symptoms of an aortic rupture include: abdominal, chest and back pain and signs of external chest injury. Because traumatic aortic transection is a life-threatening condition, treatment is needed immediately. The endovascular treatment is done in some suitable cases.^2^ Open surgery is offered to those who are unsuitable for endovascular procedure. No time should be wasted for diagnostic investigations other than computer tomography of the thorax as this investigation will give the maximum information for planning a surgical intervention.

## Case Presentation

**Figure 1:**
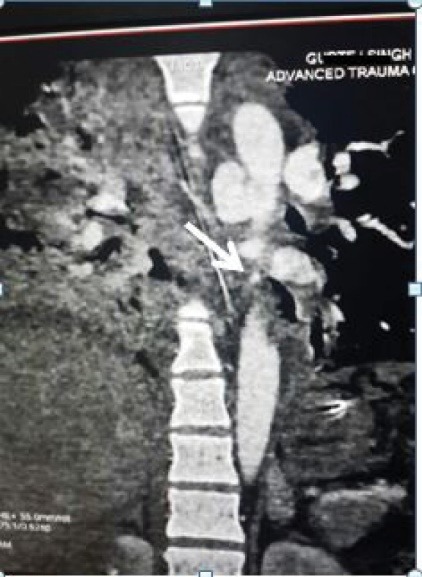
CT scan showing dissection of thoracic aorta in sagittal plane (arrow).

**Figure 2:**
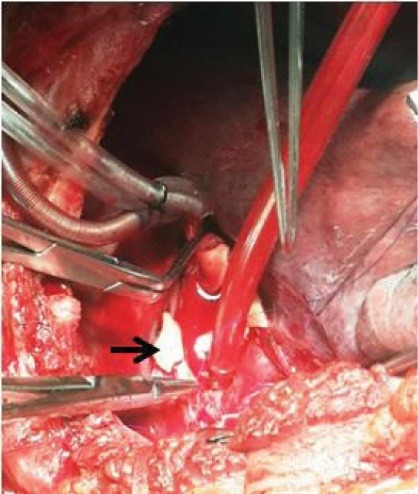
Intraoperative findings showing transected aorta (arrow).

**Figure 3:**
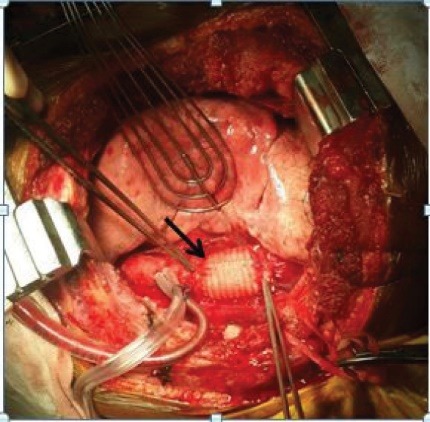
Interposition Dacron tube graft between the transected aorta (arrow).

A 29 years old male patient was taken to our emergency department within one hour of following motor vehicle accident. He complained of severe chest and back pain. On examination he was alert, conscious with pulse rate of 134 per minute, blood pressure of 86/64 mm/Hg, the extremities were cold and clammy. On auscultation, the heart sounds were audible without any adventitious sounds. The breath sounds were diminished in the left lower chest. A chest x-ray was done which revealed multiple rib fracture with mediastinal widening and left pleural effusion. In view of excruciating chest and back pain with features of hemorrhagic shock, a suspicion of aortic injury was made and hence an urgent emergency department CT scan was done after that the patient was shifted quickly to the emergency operation theater without waiting for the reports and the CT film. While shifting to the operation theater the film was obtained on the operation theater console which revealed a contained ruptured aortic rupture immediately below the level of isthmus (Figure 1). An intervention cardiologist opinion was sought for endovascular stenting but in view of lacerated aorta involving more than 50% of its circumference at the site of injury, it was not possible and hence an urgent open surgical technique was advised. The patient was taken to operation theatre and intubated with single lung tube. He was positioned in right lateral position. A left posterolateral thoracotomy was done and the chest cavity was entered through the fifth intercostal space. A huge contained hematoma was noted. The patient was heparinized a plan to conduct surgery on left heart bypass on normothermia was decided. One of the 16Fr aortic cannula was inserted to the left superior pulmonary vein as outflow circuit and another 18Fr aortic cannula was inserted to the descending thoracic aorta just above the level of diaphragm as inflow circuit and the circuit was connected to the heart and lung machine. The bypass was initiated at flow rate of 1500 mL/min. A cross clamp was applied 2 cm below the origin of left subclavian artery and another aortic cross clamp was applied on the descending thoracic aorta just proximal to the thoracic aortic cannulation. Clots and debris were removed gently and it was observed that more than half of the circumference of the aortic lumen was torn approximately 5cm distal to the left subclavian artery (Figure 2). The margins were trimmed and a 26 mm synthetic polyester Dacron tube interposition graft was sewn to both the ends using No 5-0 polypropylene suture, de-airing was performed by releasing the distal clamp first. The patient was weaned off gradually from the left heart bypass and the rest of the procedures were conducted uneventfully. The patient responded well and is on regular follow up for the last two years and the follow up CT scan of the repair is satisfactory (Figure 3).

## Discussion

Blunt aortic injury is the most lethal injury of the thorax, of which aortic transection is one of the leading cause of death.^3^ The predisposing factors are crashes of motorcycles and aircraft, auto-pedestrian collisions, falls, and crush injury.^4^ The descending aorta is fixed to the chest wall, whereas the heart and great vessels are relatively mobile. Earlier views were that a sudden deceleration causes a tear at the junction between the fixed and mobile portions of the aorta, usually near the isthmus making it the most vulnerable site.^5^ However, injury can also occur to the ascending aorta, the distal descending thoracic aorta, or the abdominal aortafollowing trauma.^6^ The diagnosis is based on a high index of suspicion, the mechanism of injury and findings of imaging studies. Chest x-ray is usually the first investigation, a mediastinal width of more than 8 cm at the level of the aortic arch is considered abnormal and is an indication for further imaging.^7^ Following features on chest x-ray guide us regarding the aortic injuries like loss of the aorticopulmonary window, abnormality of the aortic arch, rightward tracheal shift, deviation of the nasogastric tube to the right and widening of the left paraspinal line without associated fracture.^8^ Computed tomography (CT) is now the investigation of choice.^9^ Helical CT of the thorax is more sensitive for blunt aortic injury than angiography.^10^ Most blunt aortic injury patients are managed with endovascular stents while those not suitable are considered for open surgery. Our patient was a young individual who suffered road traffic accident and was taken to our emergency department in altered consciousness, he was promptly resuscitated by our triage team and the computer tomography of the thorax was performed to locate the diseased segment. Prompt shift to the operation theater saved his life. Our aim to present this case is that even the deadliest of the condition can be managed if such cases are taken on the most priority basis without wasting time.

## Conclusion

Aortic transection is a real life threatening emergencies in the real world and prompt management either in the form of stent or open thoracic surgery is the only way to save the life of such critical patients.

## Ethical approval

Written informed consent was obtained from the patient for the publication of this report.

## Competing interests

All authors declare no competing financial interests exist.
